# Prognostic Value of Immune Scoring System for Colorectal Cancer Patients with Peritoneal Metastasis

**DOI:** 10.3390/medicina60122070

**Published:** 2024-12-16

**Authors:** Berke Manoğlu, Selman Sökmen, Tufan Egeli, Zekai Serhan Derici, Cihan Ağalar, Süleyman Özkan Aksoy

**Affiliations:** Peritoneal Surface Malignancy Center, Department of Surgery, Dokuz Eylul University Faculty of Medicine, 35340 Balcova, Turkey; sokmen.selman@gmail.com (S.S.); tufanegeli@gmail.com (T.E.); serhan.derici@deu.edu.tr (Z.S.D.); cihanagalar@hotmail.com (C.A.); suleyman.aksoy@yahoo.com (S.Ö.A.)

**Keywords:** colorectal cancer, immunoscore, peritoneal metastases, mGPS, CAR

## Abstract

*Background and Objectives:* There is no reliable immune scoring system that can help us predict the postoperative outcomes of colorectal cancer patients with peritoneal metastases after cytoreductive surgery. In this cohort, the aims were (1) to evaluate the postoperative morbidity, mortality and surgical oncological outcomes in colorectal cancer patients with peritoneal metastasis; (2) to compare oncological and postoperative outcomes of colon cancer patients with peritoneal metastasis and rectal cancer patients with peritoneal metastasis; and (3) to assess the prognostic value of the modified Glasgow Prognostic Score (mGPS) and the CRP–albumin ratio (CAR). *Materials and Methods*: A prospectively maintained database of 258 patients who underwent cytoreductive surgery for peritoneal metastases of colorectal origin between 2007 and 2024 was analyzed. According to the anatomical location of the primary tumor, two different groups were created: rectum cancer patients with peritoneal metastasis (Group A) and colon cancer patients with peritoneal metastasis (Group B). All standard clinico-pathological characteristics, operative findings, morbi-mortality results, and final oncologic outcomes were compared between Groups A and B. We evaluated whether CAR and mGPS could predict postoperative morbi-mortality and overall survival in the two groups or not. *Results*: No significant difference was detected between Groups A and B in terms of clinical–demographic characteristics. In both groups, the preoperative mGPS and CAR values were statistically significantly higher in those who developed postoperative high-grade complications (C-D grade III/IV) (*p* < 0.001) and those who died perioperatively (*p* = 0.001 and *p* = 0.002). *Conclusions*: In multivariate Cox analysis, the CAR was found to be an independent prognostic factor for overall survival in this cohort. CAR and mGPS predicted high-grade complications and postoperative mortality in both groups.

## 1. Introduction

According to the data of the World Health Organization, colorectal cancer is the third most common cancer in both genders in Turkey [1]. The peritoneum is the second most common metastatic site for colorectal cancer (after the liver) [2], with up to 5–8% of colorectal cancer patients presenting with synchronous peritoneal metastases at the time of initial diagnosis, and up to 5% developing metachronous peritoneal metastases during follow up [3,4,5]. Cytoreductive surgery (CRS) with hyperthermic intraperitoneal chemotherapy (HIPEC) have great potential when it comes to treating peritoneal metastases (PMs) from colorectal cancer (CRC). The median survival time exceeds 40 months, and the survival probability at 5 years may be around 40%. If patients are selected carefully by an interdisciplinary tumor board, and if their preoperative general condition is examined critically, risks of surgery regarding morbidity are acceptable and mortality is low [6,7].

Complications that develop after extended, complex surgical procedures, such as CRS, generally lead to poor oncological outcomes, decreased survival, and high costs [8]. A relatively high rate of complications can occur in this difficult area of surgical oncology. Being able to predict the possibility of serious complications in patients can reduce morbi-mortality rates and even save lives. Finding methods to predict morbidity, mortality and overall survival in the preoperative period may guide us.

Many biomarkers used to explain systemic inflammatory reactions have been analyzed so as to unveil metastatic tumor development in the lungs, breast and gastrointestinal tract [9,10]. Glasgow researchers proved that the ratio of C-reactive protein to albumin (CAR) has critically important prognostic significance [9,10,11]. The primary disadvantage of accepting this ratio as a prognostic marker is that, depending on the threshold value used, an abnormal ratio could be obtained despite the presence of one or two markers with a normal value. A simpler and better approach is to take the modified Glasgow Prognostic Score (mGPS) into consideration as the cumulative prognostic score [12]. However, the clinical use of these prognostic markers to predict the surgical morbidities, mortalities and the prognosis in patients with peritoneal metastases is limited.

It has been proven that the activation of the systemic inflammatory response has an unfavorable impact on the outcome of patients with cancer. However, the data for the morbi-mortality results and the prognostic value of these inflammatory markers, particularly in patients with colorectal peritoneal metastasis, are limited and controversial [9,10,11,12,13]. Thus, the aim of this study was (1) to evaluate postoperative morbidity, mortality and surgical oncological outcomes in colorectal cancer patients with peritoneal metastasis; (2) to compare oncological and postoperative outcomes of colon cancer patients with peritoneal metastasis and rectal cancer patients with peritoneal metastasis; and (3) to assess the prognostic value of the modified Glasgow Prognostic Score (mGPS) and CRP–albumin ratio (CAR).

## 2. Patients and Methods

In this single-center study, a prospectively maintained database of 258 patients who underwent cytoreductive surgery for peritoneal metastases of colorectal origin between 2007 and 2024 was analyzed retrospectively. Approval was obtained from the ethics committee of Dokuz Eylul University Faculty of Medicine for the retrospective analysis of patient data (Reference No: 2024/34-21). Patients with missing data were not included in the study. The data of the routine pre- and postoperative practices performed at the Colorectal and Pelvic Surgery Unit of Dokuz Eylul University General Surgery Department were used.

According to the anatomical location of the primary tumor, two different groups were created: rectum cancer patients with peritoneal metastasis (Group A) and colon cancer patients with peritoneal metastasis (Group B). Group A and Group B were compared, and it was determined that they were homogeneous (chi-square and *t*-test). These two groups were then compared in terms of gender, age, co-morbidities (Charlson’s co-morbidity index), smoking status, tumor localization, pathology, history of neoadjuvant therapy, intraperitoneal chemotherapeutic agent, the Median Peritoneal Cancer Index (PCI) score, CC score, length of hospital stay, surgical complications [Clavien-Dindo III-IV (C-DIII-IV; ‘high-grade complication’)] and mortality rates by univariate analysis. Postoperative morbidity was defined as surgery-related morbidity seen within 90 days after surgery. Morbidity classification was made according to the Clavien-Dindo (C-D) classification. We analyzed C-D III-IV patients. Postoperative mortality was defined as death occurring within 30 days after surgery. They were analyzed for overall survival rates at 1 and 3 years.

Blood tests carried out the day before surgery were used to determine the patients’ C-reactive protein-to-albumin ratio (CAR) and the modified Glasgow Prognostic Score (mGPS). The values were compared between the two groups (Mann–Whitney U test). ROC analyses were performed for CAR and mGPS. Cut-off values and sensitivity and specificity ratios were determined. We evaluated whether CAR and mGPS could predict postoperative morbi-mortality and overall survival in the two groups or not.

We tried to assess whether or not CAR and mGPS had any statistical significance in the prediction of postoperative morbidity, mortality and overall survival.

### Statistical Analysis

Statistical analysis was performed using SPSS 24.0. Categorical variables were compared among groups using the Pearson chi-square test. Continuous variables were compared by an independent-samples *t*-test. Continuous variables were expressed as medians and ranges, and categorical variables as frequencies and percentages. Univariate analysis was performed using the chi-square test, and multivariate analysis was performed using a binary logistic regression model. Survival rates were calculated using the Kaplan–Meier method and compared with the long-rank test. Multivariate analysis to identify predictors of survival was performed by constructing stepwise Cox proportional hazard models incorporating variables selected on the basis of the results of univariate analysis. *p* values < 0.05 were defined as statistically significant.

## 3. Results

Group A with the median age of 57 (range, 22 to 73) years consisted of 33 (57.9%) male and 24 (42.1%) female patients. Group B with the median age of 58 (range, 16 to 86) years consisted of 82 (48%) male and 89 (52%) female patients. The primary tumor pathology of the patients in both groups was determined as adenocarcinoma. The median values of the Peritoneal Cancer Index (PCI) of patients in Groups A and B were 11 (range, 3–24) and 9 (range, 2–23), respectively. In addition, 75% (n = 170) of the patients underwent complete cytoreduction (CC-0). The HIPEC and EPIC (early postoperative intraperitoneal chemotherapy) procedures used in both groups were similar. The clinico-demographic characteristics are shown in Table 1.

The co-morbidity rates were 39% (n = 25) and 42.4% (n = 277) in G-A, and G-B, respectively. No statistically significant differences were found between Groups A and B when they were compared in terms of gender, smoking status, co-morbidities, history of neoadjuvant treatment, histotype, CC-scores, PCI scores, length of hospital stay (LoHs) and postoperative morbidity and mortality (Table 1).

**Figure 1:**
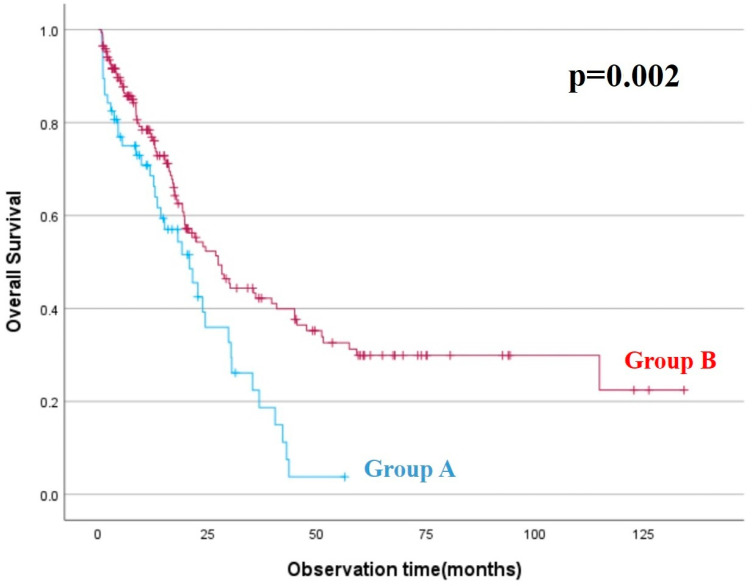
Overall survival results between Group A and Group B patients. [Log Rank (Mantel–Cox) *p* = 0.002, chi-square].

High-grade complication (C-D grade III–IV morbidity) rates in Groups A and B were 23.9% (n = 11) and 14.6% (n = 25), respectively. Perioperative mortality rates were 7% (n = 4) in G-A and 5.3% (n = 9) in G-B. The most important reason for failure-to-survive in both groups was multiple organ failure due to pleuro-pulmonary complications. There was no statistically significant difference between both groups in terms of morbi-mortality rates (Table 1). In addition, the 1- and 3-year overall survival rates of G-A and G-B patients were 68% vs. 94% and 22% vs. 42%, respectively. The overall survival of group B patients was statistically higher than group A patients (*p* = 0.002). (Table 2) (Figure 1).

There was no statistically significant difference between the preoperative mGPS and CAR values of group A and group B patients (Table 3).

In both groups, the preoperative mGPS and CAR values were statistically significantly higher in those who developed postoperative high-grade complications (C-D grade III/IV) (*p* < 0.001) and those who died perioperatively (*p* = 0.001 and *p* = 0.002) (Table 4 and Table 5).

Preoperative cut-off values of the CAR were determined as 2.56 and 4.06 for high-grade morbidity and perioperative mortality, respectively. The preoperative cut-off value of mGPS was determined as 1. Patients with values above these values had a higher risk of experiencing postoperative high-grade complications and perioperative mortality.

In multivariate Cox analysis, the CAR was found to be an independent prognostic factor for overall survival in this cohort (Table 6).

## 4. Discussion

The conventional treatment for colorectal PM is radical treatment combining complete CRS followed by intraperitoneal chemotherapy, either HIPEC or early postoperative intraperitoneal chemotherapy. These treatment procedures have strongly changed the prognosis of patients with PM from CRC. However, this aggressive locoregional therapy can be proposed only to patients highly recommended based on their general status, the extension of the peritoneal disease and the absence of extra peritoneal disease [14,15,16]. The management of PM patients is highly difficult due to the lack of clear-cut selection criteria and treatment protocols. Centers with compelling evidence and experience evaluate these patients in their multidisciplinary councils, and a tumor board makes a professional judgment concerning the most appropriate treatment option. Variable postoperative morbidity and mortality rates of a CRS&HIPEC procedure according to the patients’ age, co-morbidities, performance status, and extent of tumor load may complicate the decision-making process [17,18,19].

In the studies conducted, when we look at the postoperative morbidity and mortality rates in patients who underwent CRS&HIPEC due to peritoneal metastasis, we see that similar values are determined. Bouquet et al. reported high-grade complications in 24% and postoperative mortality in 4% of their patients [20]. Kienmanesh et al. also reported high-grade complication and postoperative mortality rates of 39% and 2.3%, respectively [21]. Meanwhile, Goelse et al. demonstrated that high-grade complications occurred in about 40% of their patients [22]. In another multi-center study, postoperative high-grade complication rates were observed to be between 10.8% and 20% [23]. Cavaliere et al. reported high-grade complication and postoperative mortality rates of 27% and 2.7%, respectively [24]. In our study, postoperative morbi-mortality rates are similar to the literature and no significant difference was found in terms of postoperative morbi-mortality rate between the group A and group B patients (23.9% vs. 14.6% and 7% vs. 5.3%). When we looked at the overall survival between the two groups, we found that the 1- and 3-year survival rates were higher in the colon-related PM patient group (68% vs. 94%, and 22% vs. 42%). As it is known, rectal cancer has a worse prognosis than colon cancer [25]. The data in our study support this situation.

Colorectal PM patients, like other PM patients, are a patient group whose postoperative management is difficult and may be faced with high-grade postoperative morbidity and mortality due to complex CRS and existing disease burden.

One of the most common causes of postoperative morbidity is surgical site infection (SSI). Many factors such as long surgical procedures, high tumor burden and exposure to various chemotherapeutic agents increase the risk of SSI. In this case, prolonged hospital stay may lead to increased costs, high-grade morbidity, sepsis and mortality. In addition, it may cause poor oncological outcomes by prolonging the time it takes for the patient to start adjuvant treatment [26,27]. In our study, the most common cause of morbidity was SSI.

In order to ensure good treatment management and reduce postoperative morbi-mortality rates, it is very important to make patient-specific treatment plans in the preoperative period. For these reasons, we tried to determine a scoring system that would allow us to predict patients at high risk of experiencing high-grade complications and mortality in the preoperative period.

The widely used Acute Physiology and Chronic Health Evaluation II (APACHE II) and Physiologic and Operative Severity Score for the enumeration of Mortality and Morbidity (POSSUM) scoring systems are recognized as useful scoring systems that help surgeons identify patient groups at high risk of complications [28]. Another scoring system is the National Early Warning Score (NEWS II). It is an effective scoring system that provides information about postoperative morbi-mortality in oncology patients planned to undergo major surgery [29].

There are studies showing that butyrylcholinesterase (BChE) enzyme values can be used as another postoperative morbidity indicator [30,31]. The non-specific cholinesterase enzyme BChE has been linked to the development of hepatic dysfunction and, more recently, to infectious disorders and septic shock. Research on the potential use of BChE in various systemic inflammatory conditions is still underway. It has been reported that low levels of BChE after colorectal cancer surgery increase the risk of surgical site infection [32]. It is still unclear if these initial findings may be used to forecast infection following colorectal surgery.

Recently, many biomarkers used to evaluate the systemic inflammatory response have been subjected to research in order to monitor the clinical progression of various solid tumors, particularly those involving the gastrointestinal tract. Many studies reported by Glasgow researchers have proved that both mGPS and CAR are critically significant prognostic markers [33,34]. We found that mGPS and CAR yielded the best predictive accuracy to determine morbidity and mortality in colorectal peritoneal metastase patients. In this cohort of patients, CAR was confirmed to be an independently significant prognostic marker in patients with colorectal PM. A recurrence-promoting inflammatory environment in the peritoneum is a strong predictor of recurrence, indicating the presence of inflammatory infiltrates in metastatic tumoral nodules [35]. In a disease consortium, we actually confronted complex patients with scarce and insufficient data complicating the treatment decision process. Hence, immune scores reflect ‘’the final disease severity level’’ to which multiple tumor characteristics and host-related inflammatory interactions converge and can therefore become the common and unified denominator to predict treatment outcomes in PMs caused by many etiologic factors. The mGPS and CAR may be effective, practical and clinically useful prognostic factors in this respect.

These findings have several far-reaching implications for clinical practice. The incorporation of immune scores into routine clinical assessment protocol will help to decide appropriate treatment planning in the preoperative period by selecting ideal candidates for CRS. Furthermore, for patients with elevated immune scores, a protocol for the management of systemic inflammatory response based on the tumor–host interaction can be instituted via prehabilitation therapy to improve the prognosis of the patients, and non-CRS treatment methods can be preferred in cases with increased immune scores. Appropriate patient selection for the CRS&HIPEC procedure has been investigated to ensure low postoperative morbidity–mortality rates and a good overall survival rate [20,21,22,23,36]. Therefore, an immune-scoring system was developed to help multidisciplinary teams to make proper surgical judgments for the safe and effective treatment of these patients.

The key to providing the safe, effective treatment and management of this complex patient population in the future may be artificial intelligence (AI), and its subtype called deep learning (DL), which is increasingly used in medicine. The function of DL, a branch of AI, is modeled by that of the real animal nervous system: to gradually extract higher-level features, including diagnosing and classifying histology images, artificial neurons are taught to identify patterns in a small amount of data. It can assist surgeons in selecting appropriate patients and provide clues about postoperative and long-term oncological outcomes [37,38,39,40].

The authors are aware that this study has several limitations. Firstly, since it has been performed retrospectively, though scarce in number, some patients (<5%) had to be excluded from the study due to missing data. Secondly, because of the retrospective nature of the study, it was difficult to control study bias and to establish a solid ‘cause-and-effect’ relationship. Our small-scale, single-center pilot study was performed on a very limited number of patients. We thought that the predictive value of this scoring system needs to be validated by data obtained from multi-centric, multinational research studies. Although the database of this single-center prospective study has been strictly monitored and supervised by a certified surgical team and hospital bioinformatics unit since 2007, the authors are absolutely sure that many uncontrollable, confounding and compelling factors related to a wide array of issues concerning long-term clinical presentations, co-morbidities, variable and unpredictable spreads of tumor load, inherited tumor mutation statuses and anesthetic and critical care management have exerted an important impact on the study results.

## 5. Conclusions

In conclusion, CAR and mGPS make up a clinically relevant immune scoring system to reduce morbidity and mortality and to improve overall survival in these high-risk patients with colorectal peritoneal metastatic disease. CAR and mGPS are simple and powerful predictive scoring systems aiming to select effectively potential candidates for CRS&HIPEC. In cases with a high risk of postoperative morbidity, that is, in patients whose immune scores are above the threshold value, a more careful complex cancer care protocol can be created before surgery, similar to the ERAS (Enhanced Recovery After Surgery) protocol. In this way, postoperative morbidities can be avoided/decreased and timely chemotherapy can be delivered. ‘Untreated’ preoperative immunosupression, ongoing inflammation and nutritional deficiencies evidently affect the postoperative course adversely by increasing rates of morbidities, mortalities, local recurrences and distant metastases in the long term. The meticulous selection of surgical candidates constitutes the sine qua non component of management of these difficult-to-treat cancer patients. Overall patient survival can be improved by effectively diagnosing and treating complications of this challenging condition.

## Figures and Tables

**Table 1:** Demographic and therapeutic characteristics of Group A and B.

	Group A (Rectum PM) n%		Group B (Colon PM) n%		*p* Values
N Patients	57		171		
Age, Year [median (IQR)]	57 (22–73)		58 (16–86)		0.083
Sex					0.194
Male	33	57.9	82	48	
Female	24	42.1	89	52	
Co-morbidity					0.646
Yes	31	54.3	87	50.8	
No	26	45.7	84	49.2	
Smoking Status					0.287
Yes	18	31.5	44	25.8	
No	39	68.5	127	74.2	
Neoadjuvant Chemotherapy					0.006
Yes	41	71.9	87	50.8	
No	16	28.1	24	49.2	
Tumor Pathology					0.578
Mucinous adenocarcinoma	12	21	39	22.8	
Non-mucinous adenocarcinoma	45	79	132	77.2	
Completeness of Cytoreduction Score (CC Score)					0.079
0	42	77.8	128	82.6	
1–2	15	22.2	43	17.4	
Peritoneal Carcinomatosis Index (PCI)					0.337
PCI ≤ 10	27	47.3	102	59.6	
PCI 10–20	27	47.3	64	37.4	
PCI ≥ 20	3	3.4	5	3	
Median PCI (IQR)	11 (3–24)		9 (2–23)		
Hyperthermic Intraperitoneal Chemotherapy (HIPEC)					0.717
Oxaliplatin	41	70.6	118	69	
Mitomycin	10	17.2	32	18.7	
Early Postoperative Intraperitoneal Chemotherapy (EPIC)					
5-Fluorouracil	7	12.2	21	12.4	
Length of Hospital Stay [Median Day (IQR)]	17 (7–90)		13 (4–94)		0.014
High-Grade Complication (CD III/IV)					0.106
Yes	11	23.9	25	14.6	
No	46	76.1	146	85.4	
Postoperative Mortality (0–30 day)					0.329
Yes	4	7	9	5.3	
No	53	93	162	94.7	

CD III/IV: Clavien-Dindo III-IV, *p*: chi-square test, standard deviation: std, IQR: Inter Quantile Range.

**Table 2:** Overall survival results (Kaplan–Meier, *p* = 0.002).

			Mean ^a^ 95% Confidence Interval	
Group	Estimate	Std. Error	Lower Bound	Upper Bound
Group A	21.445	2.343	16.853	26.036
Group B	53.297	5.096	43.309	63.286
Overall	46.350	4.174	38.169	54.531

^a^ Estimation is limited to the largest survival time if it is censored.

**Table 3:** Comparison of median mGPS and CAR values of groups A and B. (chi-square test, Mann–Whitney *U* test).

	mGPS Median (Ranging)	CAR Median (Ranging)
G-A	1 (0–2)	2.7 (0.04–79)
G-B	0 (0–2)	2.52 (0.09–108)
*p* Values	0.379	0.531

mGPS: modified Glasgow Prognostic Score; CAR: C-reactive protein–albumin ratio. G-A: Group A, G-B: Group B.

**Table 4:** ROC-analyzed CAR and mGPS with postoperative morbidity.

Risk Factor	AUC (95%)	Cut-Off	Sensivity %	Specificity %
CAR	0.730 (0.647–0.814)	2.56	77.8	44.8
mGPS	0.737 (0.649–0.825)	1	83.3	44.3

mGPS: modified Glasgow Prognostic Score; CAR: C-reactive protein–albumin ratio; AUC: area under curve (CAR, mGPS; *p* < 0.001).

**Table 5:** ROC analyzed CAR and mGPS with postoperative mortality.

Risk Factor	AUC (95%)	Cut-Off	Sensivity %	Specificity %
CAR	0.759 (0.622–0.896)	4.06	76.9	34.9
mGPS	0.767 (0.652–0.883)	1	92.3	47.9

mGPS: modified Glasgow Prognostic Score; CAR: C-reactive protein–albumin ratio; AUC: area under curve (CAR; *p* = 0.002, mGPS; *p* = 0.001).

**Table 6:** Cox regression analysis, CAR independent prognostic factors for overall survival.

	*p*	HR	95.0% CI	
			Lower	Upper
CAR	0.002	1.770	1.235	2.536

CAR: C-reactive protein–albumin ratio; HR: hazard ratio; CI: confidence interval.

## Data Availability

The data sets used and/or analyzed during the current study are available from the corresponding author on reasonable request.
